# Dr. Rowbotham on the Effects of a Diet of Fruit and Saccharine Food

**Published:** 1842-07-23

**Authors:** S. Rowbotham


*Effects of a Diet of Fruit and Saccharine Food.* By S. ROWBOTHAM, M. D.—In September last (1841) I met with a Mr. Fielding, who resides in John-street, Stockport, Cheshire, and he named to me the case of his child, which had then been ill eighteen months; he asserted that such a case was never heard of before, and desired me to see it. I accordingly went to his residence. On entering the house the stench was almost intolerable: this I was told had existed for some time. The child was a boy three years old. It was covered from head to foot with ulcers. Its eyes, nose, ears, and mouth, and, in fact, its whole head and face, were involved in one complete mass of fœtid running sores or ulcers. The lower parts of its body were the same; the anus and genitals were scarcely distinguishable, and almost black; it voided urine from five or six places; and so great were its sufferings in going to stool, that it was afraid to do so until the fæces involuntarily dropped from it, when it always cried in the most pitiful manner. Its little thighs seemed to be nearly separating from its body; and for more than twelve months it had never been able to sit down even upon a pillow, but always stood upon its feet, and leaned with its elbows upon the nurse, excepting at times when it was able to kneel upon a pillow. It had scarcely been able to lie in bed for the same period, in consequence of the whole of the surface of its head and face having run into one mass of ulceration; it had been perfectly blind for nearly twelve months. During the whole of this period it was never left alone for a moment, either night or day; its belly was exceedingly enlarged and very hard. Eight of the most eminent medical men had given it up as incurable. Some of them declared that no known mortal power could even *improve* its condition; but to think of a *cure* was madness. All who saw it agreed that it was out of the power of any human being to do it good; and any one who offered such a thing with the view of being paid for it, could be very little less than an impostor.

From certain views which I held upon the origin of disease, but which I need not state here, I was induced to recommend a diet consisting almost entirely of ripe fruits and honey, or sugar and treacle. The parents and friends of the child had long lost all hope of a cure; but could of course, see no harm in this. We accordingly commenced the new diet on the 13th of September last. In the morning it was supplied with some stewed fruits, mixed with sugar or honey, to its breakfast. The same to dinner, tea, and supper. It was allowed, and sometimes forced to be eating grapes, cherries, plums, apples, pears, and such other fruits as could be obtained, all the day through. A *little* other and ordinary food was *now and then* allowed; for previous to this change in its diet, it had almost lived entirely upon wheaten bread and butter, and bread made into porridge with milk. The appetite was so voracious that it was never satisfied; and the child would eat until its throat and mouth were crammed so full, that many times they were obliged to force out the food to prevent its being choked. On the 16th of the same month we thought the sores upon its back and neck were beginning to disappear. On the 23d it was very sensibly improved. On the 30th one half of its face was clear; its stools were made with ease; the urine was easily and freely discharged; it could sit in a chair, and lie comfortably in bed, and the lower parts of its body were much better.

In this way it went on daily improving, until at last its eyes opened, but they were very weak at first, and it could scarcely see anything; its sight, however, gradually improved. Before the new year came in, not a single ulcer remained upon its body; the skin became remarkably clear and fair; and the features, which for more than twelve months had been in such a state that it was impossible to do more than guess at the position of its nose and eyes, were restored to their wonted appearance. The father of the child at this time, when its eyes were yet exceedingly tender, in his anxiety to make known the result of the experiment, incautiously took it out without anything over its eyes; it thus took cold, and they closed up again for a few weeks; after which they again opened, and continued to strengthen and improve.

Thus, by the simple and even pleasant process of living for a time upon ripe fruits and saccharine matter,—as honey, sugar, and treacle, was a human being snatched from the grave, and from a state which, perhaps, no mortal was ever in before, and restored to health and life.—*London Lancet.*

